# A survey regarding orthodontic treatment among contemporary college freshmen in China

**DOI:** 10.1186/s12903-022-02388-8

**Published:** 2022-08-16

**Authors:** Tianyi Wang, Haolin Li, Xi Fan, Yongwen Guo

**Affiliations:** 1 State Key Laboratory of Oral Diseases & National Clinical Research Center for Oral Diseases, West China Hospital of Stomatology, Sichuan University, 14# Third Section, Renmin Nan Road, Chengdu, 610041 China; 2 State Key Laboratory of Oral Diseases & National Clinical Research Center for Oral Diseases, Department of Orthodontics, West China Hospital of Stomatology, Sichuan University, 3rd Section, No. 14, Renmin South Road, Chengdu, 610041 Sichuan China

**Keywords:** China, Malocclusion, Orthodontics, Dental Health Surveys, Psychology

## Abstract

**Background:**

A growing number of Chinese residents are seeking orthodontic treatment. The aim of the study was to investigate rates of orthodontic publicity, orthodontic treatment history and related factors among college freshmen in China, to preliminarily understand the current situation of orthodontic publicity and treatment in China, especially after the 2020s.

**Methods:**

Referred to previous studies, a self-designed online questionnaire of Chinese college freshmen was conducted. The questionnaire was divided into two parts, after collecting basic information, we investigated orthodontic publicity rate and orthodontic treatment history with starting age, relapse phenomenon, satisfaction score and willingness to re-treatment. Factors hindering first-time and second-time treatment were also collected. The statistical analysis was performed using the Chi-square test, t-test, Kruskal–Wallis test and logistic regression analysis.

**Results:**

The response rate was 100% and 3308 responses were included. The male to female ratio was 1524:1784 with a mean age of 18.43 years (SD 0.91 years). Statistically significant of economic administrative regions, age, major and orthodontics engagement of relatives or friends were found on prevalence and rate of orthodontic treatment (*P* < 0.05). Average opinion on orthodontic treatment was “satisfied” and most claimed a phenomenon of relapse (slight relapse: 38.22%, obvious relapse: 23.12%), where age, major and orthodontics engagement of relatives or friends showed statistically significant. Retreatment willingness was correlation with start age and relapse phenomenon. For those who refused first-time treatment, except for good tooth arrangement condition, pain, long-time span, and cost weighted, while for those who refused re-treatment, the fair of discomfort and long-time span ranked at the top.

**Conclusions:**

In contemporary China, the prevalence and rate of orthodontic treatment had been greatly promoted. Earlier age for receiving orthodontic treatment was also discovered. Subjective factors regarding psychology, self-requirement affected the need of orthodontic treatment. Contemporary Chinese college freshmen had a high ability of judging dental alignment, while improve of overall orthodontic care level is still required.

## Background

Malocclusion, which is defined as one of the three major oral diseases by the World Health Organization, has a high prevalence with wide age distribution in the population [1]. It affects not only the oral health and function, but also the dento-facial aesthetics, mental health, as well as oral health-related quality of life (OHRQoL) [2]. Orthodontic treatment has been suggested to alleviate the symptoms of malocclusion and prevent the occurrence of malocclusion [3, 4]. Also, extensive evidence claimed that orthodontic treatment has a positive impact on patients’ OHRQoL, including in the dimensions of emotional and social well-being [5].

Over the past two decades, with the implementation of various economic policies, the living conditions of Chinese residents have been greatly improved, thus enriching the consumption structure. After guaranteeing basic needs, contemporary Chinese people pay more attention to the satisfaction of internal needs and the pursuit of beauty, among which improving oral and maxillofacial appearance, health and function are of great demand. As a result, Chinese people have taken another step up the ladder of awareness and demand for oral health, which also indirectly stimulate the rapid development of orthodontic treatment related fields [6, 7].

A glance of cross-sectional surveys of orthodontic-related conditions led us to understand that orthodontic development in China has not been promising over the past decade. In 2006, 17.9% of the 500 non-medical college students in Dalian, China, knew nothing about orthodontic treatment [8], and the orthodontic publicity rate of college students in Zhengzhou was 54.3% in 2012 [9]. In 2013, the proportion of college students in Xinjiang who didn’t literally know orthodontics was as high as 24.08% [10]. In terms of orthodontic treatment rate, only 7.5% of non-medical college students in Dalian received orthodontic treatment in 2006 [8]. The survey of primary schools, middle schools and college students in Beijing, capital city of China, in 2008 showed that 19.0% participants had received or were undergoing orthodontic treatment [11]. Even in the 2010s, the orthodontic treatment rate in many areas still remained below 15% [12, 13]. These data strongly proved that in China, 10 or 20 years ago, the orthodontic publicity rate and orthodontic treatment rate were not that optimistic, and regional differences were also obvious. When it comes to 2020s, statistics of the level of orthodontic publicity rate and the change of orthodontic treatment awareness as well as the relevant influencing factors are still in lack.

At present, it is suggested that adolescence is the optimal time for orthodontic treatment, most teenagers with deciduous and young permanent dentition will seek orthodontic treatment during middle and high school period [14]. Therefore, the college freshmen, who often come from diversified and relatively independent social environment all around China, would better reflect the situation of orthodontic publicity and treatment for adolescents or earlier age groups all over the country. What’s more, their urgently need to establish self-esteem, including the requirements for improving oral and maxillofacial appearance, and abilities to think independently provide a reliable source for the analysis of relevant psychological factors such as uptake of orthodontic treatment and satisfaction of previous orthodontic treatment.

Hence, to understand the status of orthodontic publicity and treatment among contemporary college freshmen in China, especially after the 2020s, we designed this cross-sectional study with the hope that the findings will provide guidance for the prevention and treatment of malocclusion and laying the foundation for in-depth research and survey. In addition to analyzing the data collected in this study, we also reviewed the previous studies in China and relevant data in the same period abroad, to provide insights for improvement of overall level of orthodontic publicity and treatment in contemporary China.

## Material and methods

This study was designed to understand the current situation of orthodontic publicity and treatment in China, especially after the 2020s, and was approved by the ethics committee of West China Hospital of Stomatology, Sichuan University.

A questionnaire was designed with reference to previous study [15, 16] and clinical work experience. The questionnaire was divided into two parts. The first part was the collection of participants' basic information, including age, gender, major and economic administrative regions (Table 1). The second part was intended to investigate orthodontic publicity rate, together with orthodontic treatment rate and related factors. All participants needed to answer whether they had heard of orthodontics, whether their relatives and friends were engaged in orthodontics related fields, whether they had had orthodontic treatment, and factors hindering orthodontic treatment or orthodontic retreatment, 7 options related to tooth arrangement, economic situation, and psychological factors by consulting different researches [17–19] were offered (Fig. 2). Also, an option “other” was added for participants to fill in the answer that was more appropriate. For participants with orthodontic treatment history, they needed to further answer the starting age of treatment, relapse phenomenon, satisfaction score and willingness to re-treatment. The starting age of treatment was classified according to the growth process to “At the age of 3–6/7–11/12–15/16 or later.

**Table 1:** Demographics of participants returning questionnaires (n = 3308)

	N	%
Sex (N_a_ = 3308)		
Female	1784	53.93
Male	1524	46.07
Major (N_a_ = 3308)		
Science	375	11.34
Engineering	887	26.81
Medicine	649	19.62
Agronomy	99	2.99
Literature	242	7.32
History	149	4.50
Philosophy	150	4.53
Economics	209	6.32
Management	210	6.35
Law	167	5.05
Education	88	2.66
Art	83	2.51
Economic Administrative Regions (N_a_ = 3308)		
The eastern region	1032	31.20
The central region	493	14.90
The northeast region	191	5.77
The western region	1533	46.35
Hong Kong, Macao, Taiwan and Overseas	59	1.78

Age: mean 18.43 years; SD 0.91 years; range 16.00–25.00 years

The options design of all questions is reflected in Tables 1, 2 and Fig. 2. Comprehensive description and non-professional terms were used to ensure full understanding of all participants. The questionnaire was distributed mainly among universities in West China and the original data were collected online through Sojump, a Web-based survey tool, and spread through online chat groups. The criteria for the respondents of this survey were freshmen, regardless of gender and region. The answer collection started from September 27, 2021 and lasted for 2 weeks. All participants were given informed consent to the collection and use of information.

**Table 2:** Participants’ orthodontic background and relevant conditions

	N	%
Have you ever heard of orthodontics? (N_a_ = 3308)		
Yes	2922	88.33
No	386	11.67
Do you have any relatives or friends engaged in orthodontic-related fields? (N_a_ = 3308)		
Yes	1071	32.38
No	1848	55.86
Not sure	389	11.76
Do you have experience of orthodontic treatment? (N_a_ = 3308)		
Yes	1609	48.64
No	1699	51.36
If yes, when did you start orthodontic treatment? (N_a_ = 1609)		
At the age of 3–6	211	13.11
At the age of 7–11	522	32.45
At the age of 12–15	567	35.24
At the age of 16 and later	309	19.20
Are you satisfied with the results of orthodontic treatment? (N_a_ = 1609)		
Very dissatisfied	89	5.53
Dissatisfied	280	17.40
Satisfied	801	49.78
Very satisfied	439	27.29
Do you think your teeth have a phenomenon of relapse? (N_a_ = 1609)		
Not sure	120	7.46
No relapse	502	31.20
Slight relapse	615	38.22
Obvious relapse	372	23.12
If your orthodontic treatment is now over, is there a need to retreat? (N_a_ = 1609)		
Unnecessary or dispensable	642	39.90
Necessary	967	60.10

### Statistical analysis

SPSS statistical software (version 25, IBM, Armonk, NY) was used to summarize the original data and carry out correlation statistical analysis. Descriptive analyses of the answers to each question were conducted, together with the frequencies of each answer.

**Fig. 1:**
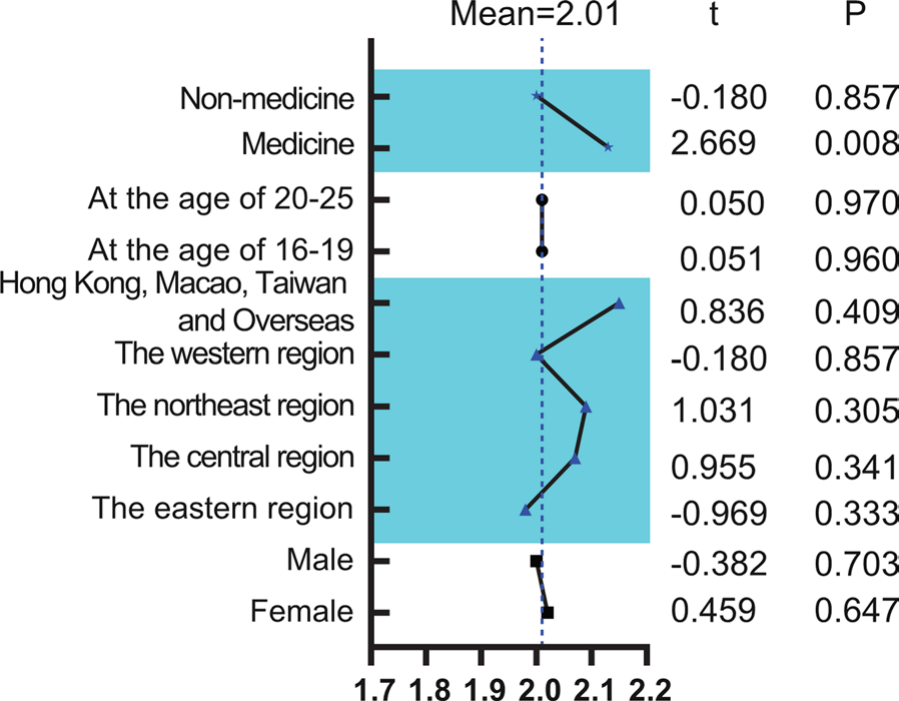
Score of satisfaction of orthodontic treatment of various groups with a mean score of 2.01. Scores from 1–4 referred to “very satisfied” to “very dissatisfied”. A mean score of 2.01 indicates an average opinion of “satisfied”. ** Indicate a significant difference at *P* = 0.01 level

**Fig. 2:**
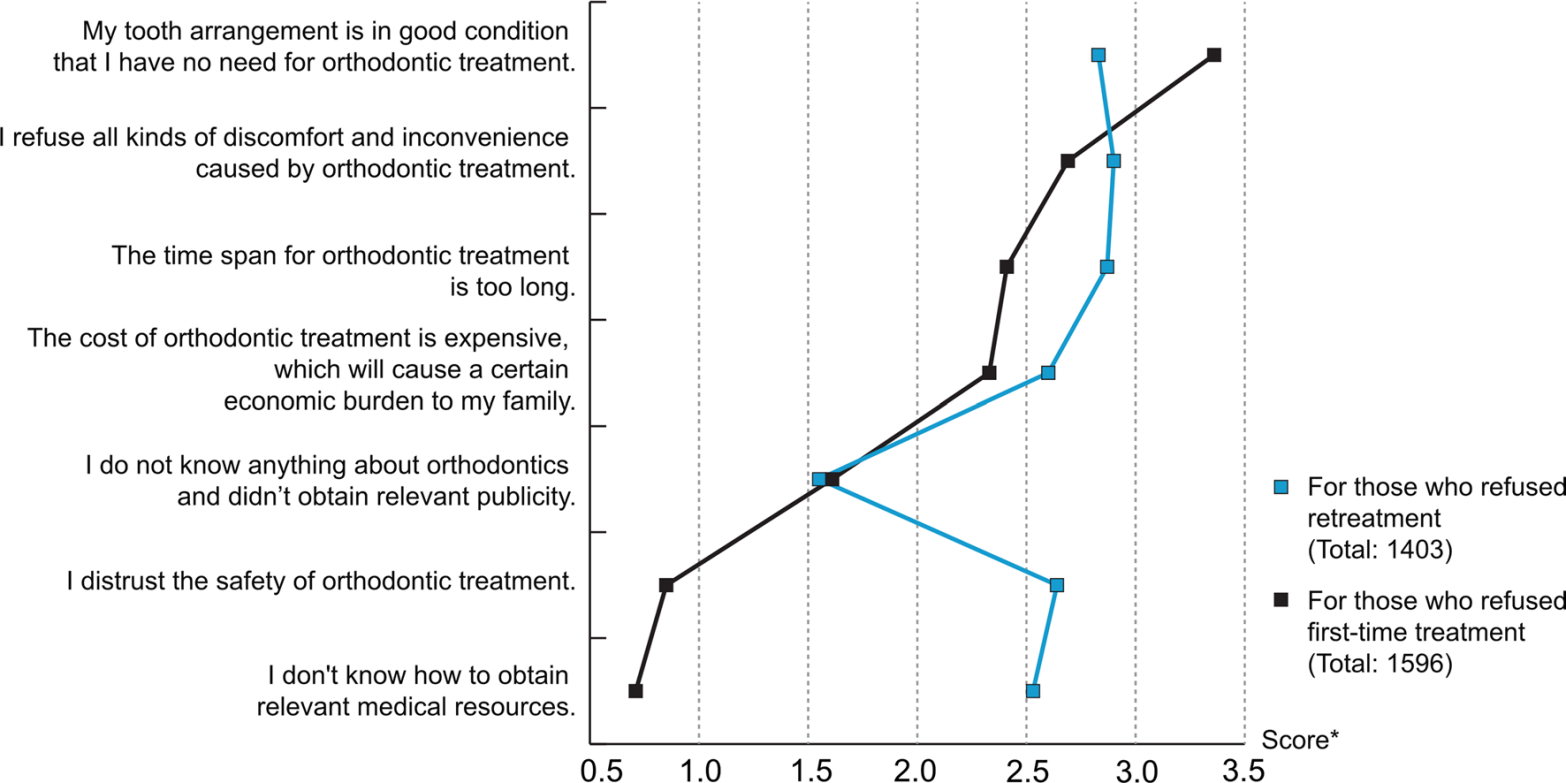
Factors hindering participants’ practical action for first-time treatment and re-treatment. 7 opinions provided were shown in the figure without the opinion of “others”. *Score = (Σ frequency × weight)/person times filled in this question, whose weight ranging from 8 to 1

Chi square test was used to analyze the demographic differences of orthodontic publicity rate, orthodontic treatment rate and phenomenon of relapse. Chi-square *P* value and χ^2^ were listed in Tables 3 and 4. T-test counted the demographic differences of satisfaction with t and *P* value shown in Fig. 1. After analyzing correlation analysis of orthodontic related factors, the correlation between these variables were all less than 0.7, proving their independence from each other, and therefore all were included in the latter stage of the logistic regression analysis (Table 5). Logistic regression analyzed the orthodontic publicity rate, orthodontic treatment rate, factors affecting the willingness to re-treatment in more detail, which was firstly selected by correlation analysis (Table 6). The value of each factor hindering participants’ practical action for first-time treatment and re-treatment were calculated by a formula offered by Sojump: score = (Σ frequency × weight)/person times filled in this question which ranging from 8 to 1 (Fig. 2). A *P* value of < 0.05 was considered statistically significant.

**Table 3:** Frequency of orthodontics publicity and orthodontic treatment history by backgrounds of participants

	Have you ever heard of orthodontics?			Chi-square *P*	χ^2^	Do you have experience of orthodontics treatment?			Chi-square *P*	χ^2^
	Yes N (%)	No N (%)	Total N (%)			Yes N (%)	No N (%)	Total N (%)		
Gender				0.072	3.230				0.564	0.333
Female	1594 (89.35%)	190 (10.65%)	1784 (53.93%)			876 (49.10%)	908 (50.90%)	1784 (53.93%)		
Male	1328 (87.14%)	196 (12.86%)	1524 (46.07%)			733 (48.10%)	791 (51.90%)	1524 (46.07%)		
Economic Administrative Regions				0.000**	28.070				0.000**	56.439
The central region	442 (89.66%)	51 (10.34%)	493 (14.90%)			183 (37.12%)	310 (62.88%)	493 (14.90%)		
The northeast region	161 (84.29%)	30 (15.71%)	191 (5.77%)			87 (45.55%)	104 (54.45%)	191 (5.77%)		
The eastern region	902 (87.40%)	130 (12.60%)	1032 (31.20%)			466 (45.16%)	566 (54.84%)	1032 (31.20%)		
The western region	1376 (89.76%)	157(10.24%)	1533 (46.35%)			840 (54.79%)	693 (45.21%)	1533 (46.35%)		
Hong Kong, Macao, Taiwan and Overseas	41 (69.49%)	18 (30.51%)	59 (1.78%)			33 (55.93%)	26 (44.07%)	59 (1.78%)		
Age				0.010**	6.551				0.003**	8.834
16–19	2519 (88.92%)	314 (11.08%)	2833 (85.64%)			1348 (47.58%)	1485 (52.42%)	2833 (85.64%)		
20–25	403 (84.84%)	72 (15.16%)	475 (14.36%)			261 (54.95%)	214 (45.05%)	475 (14.36%)		
Major				0.000**	21.842				0.021*	5.319
Medicine	539 (83.05%)	110 (16.95%)	649 (19.62%)			342 (52.70%)	307 (47.30%)	649 (19.62%)		
Non-medicine	2383 (89.62%)	276 (10.38%)	2659 (80.38%)			1267 (47.65%)	1392 (52.35%)	2659 (80.38%)		
Do you have any relatives or friends engaged in orthodontics?				0.000**	44.624				0.000**	157.621
No	1670 (90.36%)	178 (9.63%)	1848 (55.86%)			732 (39.61%)	1116 (60.39%)	1848 (55.86%)		
Not sure	305 (78.41%)	84 (21.59%)	389 (11.76%)			195 (50.13%)	194 (49.87%)	389 (11.76%)		
Yes	947 (88.42%)	124 (11.58%)	1071 (32.38%)			682 (63.68%)	389 (36.32%)	1071 (32.38%)		
Total	2922 (88.33%)	386 (11.67%)	3308 (100.00%)			1609 (48.64%)	1699 (51.36%)	3308 (100.00%)		

N = number in the group

Chi-square *P* = two-sided significance of difference between age groups assessed by the Pearson Chi-square test

**Means *P* < 0.01, *means *P* < 0.05

**Table 4:** Frequency of self-precepted relapse phenomenon by backgrounds of participants with orthodontic treatment history

	Relapse phenomenon				*P* value^a^
	Obvious N (%)	Slight N (%)	No N (%)	Total N (%)	
Gender					0.253
Female	211 (26.02%)	335 (41.30%)	265 (32.68%)	811 (54.47%)	
Male	161 (23.75%)	280 (41.30%)	237 (34.95%)	678 (45.53%)	
Economic Administrative Regions					0.007**
The central region	51 (31.48%)	72 (44.44%)	39 (24.08%)	162 (10.88%)	
The northeast region	18 (22.78%)	32 (40.51%)	29 (36.71%)	79 (5.31%)	
The eastern region	117 (27.27%)	180 (41.96%)	132 (30.77%)	429 (28.81%)	
The western region	180 (22.78%)	317 (40.13%)	293 (37.09%)	790 (53.06%)	
Hong Kong, Macao, Taiwan and Overseas	6 (20.69%)	14 (48.28%)	9 (31.03%)	29 (1.94%)	
Age					0.350
16–19	306 (36.82%)	100 (12.03%)	425 (51.14%)	831 (55.81%)	
20–25	66 (10.03%)	515 (78.27%)	77 (11.70%)	658 (44.19%)	
Major					0.365
Medicine	76 (24.52%)	141 (45.48%)	93 (30.00%)	310 (20.82%)	
Non-medicine	296 (25.11%)	474 (40.20%)	409 (34.69%)	1179 (79.18%)	
Do you have any relatives or friends engaged in orthodontics?					0.021*
No	187 (27.66%)	281 (41.57%)	208 (30.77%)	676 (45.40%)	
Not sure	29 (16.11%)	88 (48.89%)	63 (35.00%)	180 (12.09%)	
Yes	156 (24.64%)	246 (38.86%)	231 (36.49%)	633 (42.51%)	
Total	372 (24.98%)	615 (41.30%)	502 (33.71%)	1489 (100.00%)	

N = number in the group

^a^Kruskal–Wallis test *P* value

**Means *P* < 0.01, *means *P* < 0.05

**Table 5:** Correlation analysis of orthodontic related factors

	Start age of orthodontic treatment	Relapse phenomenon	Degree of satisfaction	Willingness of retreatment
Start age of orthodontic treatment	1			
Relapse phenomenon	0.018	1		
Degree of satisfaction	0.001	− 0.037	1	
Willingness of retreatment	0.130**	0.132**	− 0.020	1

**At level 0.01 (two tail), the correlation is significant

**Table 6:** Logistic regression analysis on the influencing factors of orthodontic retreatment willingness

	Willingness of retreatment	
	*P* value	OR (95% CI)
Start age of orthodontic treatment^a^		
At the age of 7–11	NS	–
At the age of 12–15	0.00**	0.534 (0.382–0.746)
At the age of 16 and later	0.00**	0.515 (0.357–0.743)
Relapse phenomenon^b^		
Not sure	0.00**	0.446 (0.297–0.669)
Slight relapse	NS	–
Obvious relapse	0.00**	1.781 (1.331–2.384)

*nd* the foreign contemporaneous literature

**Means *P* < 0.01, *means *P* < 0.05, NS means *P* > 0.05

^a^Compared with “At the age of 3–6”

^b^Compared with “No relapse”

## Results

A total of 3336 questionnaires were collected through online distribution. After screening of age and content, 3308 questionnaires were finally included in the statistical scope. Among the 12 questions given, the answer rate was 100% at the participant level.

### Collection of basic information

Basic information of participants was shown in Table 1. The average age of 3308 participants was 18.43 ± 0.91 years, and the proportion of male and female participants was 46.07% and 53.93%. Table 2 displayed raw data of the participants' orthodontic background and relevant conditions. Most of the 3308 freshmen surveyed had heard of "orthodontics" (88.33%), and more than half of the participants had no relatives or friends engaged in orthodontics related fields (55.86%). Participants who had ever received orthodontic treatment counts for 1609 (48.64%).

For those 1609 participants who had received orthodontic treatment, a large proportion of them began their orthodontic treatment at the age of 7–11 years (32.45%) and 12–15 years (35.24%). A half of them considered the treatment as “satisfied” (49.78%). Most of the participants thought their teeth don’t relapse or relapse at a slight degree, and 60.10% of them thought it was necessary for an uptake of re-treatment.

### Orthodontic publicity and prevalence of orthodontic treatment history

Chi square analysis of Table 3 showed that there exist significant statistical differences both in the orthodontics publicity and treatment history among freshmen of economic administrative regions, age, major and orthodontics engagement of relatives or friends (*P* < 0.05). Orthodontic publicity rates were higher among freshmen from the mainland, 16–19 age group and non-medical majors, but orthodontic treatment rates were higher among college freshmen from Hong Kong, Macao, Taiwan and Overseas, 20–25 age group and medical majors. For those who had relatives or friends engaged in orthodontic-related fields, they enjoyed both high rate of orthodontic publicity and orthodontic treatment history.

### Satisfaction, phenomenon of relapse and willingness of re-treatment

For results of satisfaction, 1–4 points were assigned to "very satisfied", "satisfied", "dissatisfied" and "very dissatisfied", that was, the lower the score, the higher the participant's satisfaction with orthodontic treatment. In Fig. 1, the average score of satisfaction of 1609 participants were 2.01, One sample t-test showed major was the only related factor of orthodontic treatment satisfaction that participants of medicine were more dissatisfied with the treatment results.

After exclusion of 120 participants who chose “unsure”, 41.30% of the participants thought they had “slight relapse”, while 24.98% thought they had “obvious relapse”. Economic administrative regions and orthodontics engagement of relatives or friends all showed statistically significant with self-precepted phenomenon of relapse (Table 4).

Correlation analysis showed that start age of orthodontic and relapse phenomenon had significant impacts on the willingness to re-treat (Table 5). In Table 6, participants who started treatment between the age of 12–15 or at 16 or later were less likely to choose to treat again (OR: 0.534, 95% CI: 0.382–0.746 and OR: 0.515, 95% CI: 0.357–0.743). Those who thought their teeth had obvious relapse were more likely to uptake re-treatment (OR: 1.781, 95% CI: 1.331–2.384), while participants who were uncertain about relapse had lower willingness to retreat (OR: 0.446, 95% CI: 0.297–0.669).

### Hindering factors for orthodontic treatment/re-treatment

Factors hindering participants’ action for orthodontic treatment/re-treatment were collected as Fig. 2. For those who refused first-time treatment, except for good tooth arrangement condition, pain, long-time span, and cost weighted. While for those who refused re-treatment, the fair of discomfort and long-time span ranked at the top.

## Discussion

### Promotion of orthodontic publicity rate and orthodontic treatment rate in China

Compared to the data of non-heard of orthodontics found about 10 years ago (17.9–24.08% with regional differences) [8, 9], our survey showed 11.67% of the participants had not heard of orthodontics. Whether or not they have friends or relatives in the orthodontic profession, freshmen could access to a high rate of orthodontic publicity through various means. Previous studies had shown that the orthodontic treatment rates in different countries vary from 5 to 60%, which was closely related to national living consumption level and relevant policies [19, 20]. With the strengthening of orthodontic popularity and the improvement of living standards, the rate of orthodontic treatment of contemporary college freshmen in China had been significantly improved (over 50%) compared to an orthodontic treatment-receiving rate of less than 20% about ten years ago [12, 13]. Nevertheless, consideration should be taken that some foreign governments could reimburse most of the orthodontic treatment expenses for patients with malocclusion who met certain conditions, or provide orthodontic treatment for free, which greatly improved the orthodontic treatment rate and control of malocclusion [19].

### Who were more likely to receive orthodontic treatment?

With the increase of age, the proportion of reception of orthodontic treatment increased. Previous studies showed this rate of undergraduates in science, literature, art, sports and medicine did not have statistical difference [13], while statistical significant of medical and non-medical on orthodontic treatment rate was found in this study. The high demand of medical students for their orofacial function and appearance may contribute. Women were more likely to receive orthodontic treatment, which was statistically significant [19]. However, the data of this study showed gender had no such essential role in the acceptance of orthodontic treatment. This may be related to the high requirements of contemporary Chinese college students neither female or male, especially the high standards of perceptual needs such as experience and comfort.

Many other factors contributed as obstacles to orthodontic treatment uptake. Those married and with low impact of OHRQoL were more likely to refuse orthodontic treatment [21], while choice on this issue did not produce statistically significant differences with those from high and low socioeconomic classes [21]. Although people from lower socioeconomic classes may not have high demands on their dento-facial aesthetics, it was also demonstrated that low socioeconomic class was linked to a greater burden of oral disease and oral health care needs in numerous developed counties [22]. Hence, it needs to be further explored to understand the detailed factors both prevent and promote the choice of orthodontic treatment in low socio-economic groups. In addition, the inevitable orthodontic contraindications during pubertal development were also a major obstacle to orthodontic treatment in adolescent population. As iatrogenic risk of orthodontics on the periodontium could not be ignored, the orthodontic forces could aggravate the symptom of patients with periodontitis even with good oral hygiene [23–25].

In the present study, differences were noticed in orthodontic publicity and treatment rate of all regions in China mainland, inferring there existed a long process from "hearing about orthodontics" to “receiving orthodontic treatment" except for those who do not need. Wen et al. found that 53.31% of undergraduates with friends who had a history of orthodontic treatment thought they need orthodontic treatment [13]. This indicated the satisfaction of relatives and friends with orthodontic treatment experience or whether relatives and friends were engaged in orthodontic related fields may play a further essential role, which helped college students who did not understand the purpose and process of orthodontic treatment realize the advantages of orthodontic treatment more [26], the similar results were also observed in our study.

### Chinese teenagers began to receive orthodontic treatment earlier than before

Although more than half of the respondents received orthodontic treatment during permanent dentition, we found that the proportion of orthodontic treatment in deciduous and mixed dentition stage was significantly higher (45.56%) than that 20 years ago, as a study in 2003 showed that only 7.89% participants started treatment in the deciduous tooth stage and 10.62% in the mixed dentition stage [27]. On one hand, the younger age of receiving orthodontic treatment found in the present study owns to the specific subjects of college freshmen we chose. On the other hand, it indicated Chinese parent’s awareness had been promoted on children’s oral health and beauty. Both expansion of relevant external medical information and the absent of objective suggestions of formal medical institutions would help aggravate the anxiety of contemporary Chinese parents about their children's oral health. Hence, there is a need for popular science of orthodontic knowledge. For instance, early orthodontic treatment may act as Janus face that apart from improving oral hygiene habits and addressing occlusal issues, it also raises risk on abnormal alveolar bone and root development [14, 28]. Moreover, normal occlusal development during mixed dentition should be noted, thus enhancing parent to judge the need for early orthodontic treatment [14].

### Satisfied, while more self-evaluation of relapse

Patients' satisfaction after orthodontic treatment has a great relationship with the clinical level of orthodontists, which also reflects the degree of promotion and perfection of the national orthodontic industry in the bio-psycho-social medical model to a certain extent [18, 29]. Cost, specific types of malocclusions, time span, occlusion after treatment, tooth alignment, tooth extraction and relapse phenomenon all affected the overall satisfaction of patients with orthodontic treatment [30]. A clinical survey on the average age of 16.2 years in 2007 showed that respondents generally had high satisfaction with the doctor-patient relationship. While with the increase of age, the lower satisfaction level occurred, and the satisfaction level of men was higher than that of women [31]. On the whole, the average score of 2.01 in our study represented that the participants were "satisfied" with orthodontic treatment, and the level of orthodontic treatment in China had developed in a balanced way, and the functional and psychological needs of men and women with different gender and age levels had been taken into account. The average score of satisfaction of medical freshmen was 2.13, which more inclined to the results of "unsatisfactory" orthodontic treatment, while non-medical freshmen tended to be more satisfied. This is also a reflection of the high expectations of medical students.

After orthodontic treatment, the trend of teeth and jaws returning to their original position is called relapse, which may happen due to a variety of factors such as unregulated retaining, insufficient retention time, poor occlusal relationship, occlusal trauma, third molars eruption and bad oral habits that were not completely removed [32]. A survey of 476 patients in retention period in 2003 showed that the relapse rate was 7.6% [33]. Tan et al. showed that 56 of the 603 patients surveyed had relapse (9.29%), and there was a significant difference in age, but no gender [34]. While in our study, the proportion of patients who thought they had a phenomenon of obvious relapse were 23.12%, which was statistically related to economic administrative regions and orthodontics engagement of relatives or friends. This suggested that the level of socio-economic development and the surrounding atmosphere can greatly influence results of orthodontic patients' self-precepted relapse. However, further survey regarding orthodontics patients seeking for retreatment.

Patients' satisfaction with orthodontic treatment largely depended on the level of relapse and expectation of stability [35]. At present, many studies showed that lifelong retention was preferred [15], but the survey conducted by Lasance et al. showed that only one third of patients were willing to performing lifelong retention, while most patients thought that 1–10 years was enough [15]. The promotion of lifelong retainer wearing needed not only the efforts of patients, but also the participation of orthodontists [36]. Regular follow-up should be given a certain degree of attention.

### Whether re-treat or not?

After the relapse of malocclusion, some patients will choose to receive re-treatment, whose causes included such as non-extraction of extraction cases, loss of anchorage after tooth extraction, single jaw correction, termination of treatment caused by distrust [32]. In the investigation performed by Tan et al. mentioned above, 18 of the 56 relapse cases chose re-treatment (32.14%) [34]. The psychological research of Wang et al. showed that the first treatment of patients with re-treatment history was mainly suggested by their parents and doctors, while the choice of re-treatment was mostly from their own will, who might have higher requirements for maxillofacial aesthetics and paying more attention to neater dentition [37].

Relatively speaking, the proportion of patients in our study who thought they need re-treatment was high, which was related to the start time of treatment and self-evaluation of relapse. As after deciduous tooth stage, unpredictable dental and maxillofacial development as well as bad oral habits may be one of the risk factors for the high willingness of re-treatment of patients who received primary orthodontic treatment at early age. Similarly, patients who found occurrence of serious relapse could naturally strengthen their willingness of retreatment. It was worth noting that patients who were uncertain about the relapse level were more inclined not to carry out secondary treatment, which reflected there existed psychological refusal of some patients. Subsequent statistics on "factors hindering re-treatment" showed that although some patients did had objective needs for re-treatment, they subjectively refused it due to discomfort, inconvenience, long time span and distrust of safety suffered in first treatment. In contrast, the economic factors previously valued had become less important obstacles. Surveys showed that the complications in the treatment process included sore mouth and gingivitis [18]. In daily life, pain, difficulty in sleep and talk, embarrassment in eating, reluctance to laugh, and even low self-esteem and depression had all become important factors affecting the mentality of patients [18]. According to the views collected on Twitter and Instagram related to orthodontic treatment, the proportion of positive attitudes was less than 50%, "braces" became the word most concerned by patients with both positive and negative attitudes, while patients with negative attitudes paid more attention on "orthodontist" and "appointment" than those with positive attitudes [17]. Thus, it could be seen that patients' attitudes towards orthodontic treatment was affected by a variety of external and internal factors, noting that special mental attention should be paid to self-esteem and psychology in the process of orthodontic treatment.

This study was not without its limitations. Uneven geographical distribution of participants was found. Also, background involving the relevant situation of the respondents' family background was vacant. The investigation on the participants who had undergone orthodontic treatment was also lack, for example, we did not investigate the type of appliance and time span of retention, which were believed to significantly affect orthodontic treatment satisfaction and relapse. In addition, we did not achieve the unity of online questionnaire and offline inspection, thus making results relatively subjective, which would also affect the accuracy of results to some extent.

## Conclusions

By investigating the orthodontic publicity and orthodontic treatment-related information of Chinese contemporary college freshmen, this study preliminarily understood the current situation of orthodontic publicity and treatment among contemporary college freshmen in China, especially after the 2020s. The relevant conclusions were summarized as follows:

1. In contemporary China, the prevalence of orthodontics and the rate of orthodontic treatment had been greatly promoted, and the age of Chinese adolescents receiving orthodontic treatment had been much earlier than before. However, compared with some countries, China's subsidy policies for orthodontic treatment had defects.
2. The need of orthodontic treatment among college freshmen in China was greatly affected by subjective factors such as psychological maturity, self-requirements for career development and the influence of surrounding people. However, different from most foreign studies, gender did not become a relevant factor of uptake of orthodontic treatment. Among factors hindering the orthodontic treatment, fear and other subjective factors were more essential than objective economic factors. Especially for those who had received orthodontic treatment before, psychological fear and distrust were more obvious.
3. Although satisfied with the orthodontic treatment results, the high proportion of those with self-perceived relapse and need for orthodontic retreatment suggests that the overall level of orthodontic treatment in contemporary China needs to be improved.
4. It is necessary to further standardize orthodontic treatment in contemporary China, especially the selection of indications for early orthodontic treatment, whose advantages and disadvantages need to be further studied in the long run.
5. This study has limitations involving uneven geographical distribution of participants, vacant investigation of family background, type of appliance and time span of retention, and lack unity of online questionnaire and offline inspection. Further studies may investigate in depth with guidance of these directions.

In conclusion, with its limitations, the present study found that in contemporary China, the prevalence of orthodontics had been greatly improved, but patients' satisfaction with the outcome was still relatively low, the self-evaluated relapse rate after treatment and the proportion requiring re-treatment were still high. These results suggested that although orthodontic treatment was widely popularized in China, the overall level of orthodontic care still needed to be improved, science education for the public was also necessary to further improve the public's understanding of orthodontics.

## Data Availability

All data generated and/or analyzed during the current study are available from the corresponding authors on reasonable request.
